# Epidermal Growth Factor Pathway Signaling in *Drosophila* Embryogenesis: Tools for Understanding Cancer

**DOI:** 10.3390/cancers9020016

**Published:** 2017-02-07

**Authors:** Jay B. Lusk, Vanessa Y. M. Lam, Nicholas S. Tolwinski

**Affiliations:** 1 Division of Science, Yale-NUS College, Singapore 138527, Singapore; jay.lusk@u.yale-nus.edu.sg (J.B.L.); ynclamy@nus.edu.sg (V.Y.M.L.); 2 Department of Biological Science, National University of Singapore, Singapore 138615, Singapore

**Keywords:** EGF signaling, *Drosophila* development, RAS, RAF, MAP kinase, MEK

## Abstract

EGF signaling is a well-known oncogenic pathway in animals. It is also a key developmental pathway regulating terminal and dorsal-ventral patterning along with many other aspects of embryogenesis. In this review, we focus on the diverse roles for the EGF pathway in *Drosophila* embryogenesis. We review the existing body of evidence concerning EGF signaling in *Drosophila* embryogenesis focusing on current uncertainties in the field and areas for future study. This review provides a foundation for utilizing the *Drosophila* model system for research into EGF effects on cancer.

## 1. Introduction to EGF Signaling

### 1.1. Function of the EGF Receptor, Its Ligands, and an Antagonist

The *Drosophila* homolog of the epidermal growth factor receptor abbreviated as EGFR is known by several names—*faint little ball* and *torpedo*—with *flb* referring to the embryonic phenotype and *torpedo* referring to an oogenesis defect observed in eggs [1], *DER* and *Ellipse* for imaginal disc and adult eye phenotypes [2,3]. It is a single-pass transmembrane receptor tyrosine kinase (RTK), and is required for a variety of roles in normal *Drosophila* embryogenesis [4,5]. The extracellular domain of EGFR contains two cysteine-rich subdomains (SII and SIV), and two extracellular cysteine-poor subdomains (SI and SIII) [4]. Following a transmembrane domain, the intracellular portion of the protein is a protein tyrosine kinase. *Drosophila* EGFR is highly conserved with vertebrate forms of EGFR, sharing 40.5% identity [6,7,8].

EGFR receives its signal from a series of different ligands, which can interact with the receptor through different molecular mechanisms [9]. These ligands include *gurken* (*grk*), *spitz* (*spi*), *vein* (*vn*), and *keren* (*krn*) [10,11,12]. Three of the four ligands, *grk*, *spi*, and *krn* show the highest homology to TGF-α whereas *vn* is homologous to *neuregulin* [10]. It was previously proposed that different EGF ligands were responsible for different EGF responses [13], however recent evidence suggests that different patterns of expression may be responsible for different EGF signaling outcomes [14]. For example, the embryonic signaling function of *vn* can be substituted for by *spi* when expressed in the endogenous *vn* pattern, showing that in some cases the identity of the ligand is not as critical compared to the ability of the receptor to bind the signal [10].

*krn* is less well-characterized than *spi*. Emerging literature appears to suggest that, aside from its alternative mechanism of transport and regulation, it is a functional homologue to *spi*, which functions redundantly with *spi* in early development [10,15,16]. However, the functional redundancy of *krn* in embryogenesis lends itself to a question with no immediately compelling answer: what selective pressure would cause *krn* to be maintained as a ligand in EGF signaling, and why would it develop an alternative cleavage mechanism to the primary ligand, *spi*.

*vn* also appears to be biochemically redundant with the TGF-α-like ligands. In fact, loss of *vn* in lateral embryonic stripes had no effect on downstream activation of ERK [17]. *vn* expression is determined by the gradient of nuclear Dorsal established by the Toll pathway [17], but *vn*’s unique function may be to activate the EGF signal in tissues where low levels of activation are required due to its weaker activation capacity than *spi* [14,18]. *spi* is able to rescue *vn* mutants and modulates its activity to roughly match the activation by *vn* [10]. Overall, these three types of ligands which serve similar functions, allow the EGF pathway to signal in a variety of conditions increasing its accuracy and specificity in effecting cellular changes in response to other developmental events.

In addition to these four extracellular ligands, Argos (*arg*) serves as a ligand antagonist by forming a clamp-like structure around *spi* and thereby inhibiting EGF signal transduction [9]. *arg* functions in the pathway through a negative feedback loop where it is produced as a result of high levels of EGFR activity [19]. *arg* is required for normal function of the EGF pathway, and loss of Arg results in excessive EGFR pathway activation and corresponding expansion of ventral cell fates [19]. *arg* therefore acts to limit long-range effects of the signal by restricting the spread of *spi* [20].

Some recent experiments suggest that the EGFR ligands are interchangeable as there is significant redundancy between ligands, similarity in mechanisms of activation, and minimal differences in regulation between the ligands [10,16,18,21]. Instead, it seems that differing quantitative transduction of signal due to differences in ligand concentration (and activation capacity), is responsible for the differentiation of EGF receiving tissues. What this means is that each ligand is capable of activating a specific amount of signal transduction, where tissues receiving the EGF signal differentiate according to the strength of signaling. There is no compelling explanation for how selective pressure led to the evolution of several redundant mechanisms. It is possible that the broad diversity and critical nature of EGF signaling both in development and in a wide range of other biological processes has influenced the development of redundant ligands with subtly differing regulatory mechanism or strength. It is also possible, especially given the similarity of *spi*, *krn*, and *grk* to TGF-α, and *vn*’s corresponding dissimilarity, that any of these ligands were co-opted from another function early in evolution, and the original function was later lost, with the ligand taking on a new role as a redundant regulator of the EGF signal. The finding that *arg* binds to the EGF receptor, not the ligand, and in a manner different from the ligands, is consistent with the idea that several ligands were co-opted from other functions while the inhibitory ligand (of which only one exists) was central to the function of the receptor prior to the evolutionary events where other ligands were co-opted [22].

### 1.2. Intracellular Regulation of EGF Ligands

EGF ligands undergo significant intracellular regulation in the process of their production. SPI is initially produced as a transmembrane pro-protein, which is then cleaved to release the extracellular signaling domain [11]. SPI is cleaved into the endoplasmic reticulum (ER), and trafficked by bulk flow to the Golgi [23,24,25]. In the absence of STAR, a ubiquitously-expressed type II single domain transmembrane protein, which binds to the inactive form of SPI, it will undergo retrograde trafficking back to the ER [26]. Formation of the STAR-SPI complex will inhibit retrograde trafficking from the Golgi to the ER [24,25]. The STAR-SPI complex is then trafficked to an endosomal compartment where Rhomboid1 (RHO-1) cleaves SPI, yielding the active ligand which can now be secreted to the extracellular space, and the STAR-SPI and RHO-SPI complexes disassociate [24,25,27,28]. Interestingly, KRN is capable of undergoing low levels of cleavage and subsequent EGF activation in the absence of *rho-1*/*star*-mediated trafficking, despite its otherwise identical function to SPI in terms of activating the EGF signaling pathway [15].

*rho-1* deserves particular attention as a regulatory protein within the EGF signaling pathway. Not only is *rho-1* very highly conserved [21], but it also has been shown to specifically act in trafficking of EGF ligands [25]. All four EGF ligands and STAR are substrates for *rho-1* mediated cleavage [24]. While the regulatory action of *rho-1* on EGF extracellular ligands is clear, it is still unclear what, if any, regulatory function RHO-1 has on STAR [25]. Both *star* and *spi* are ubiquitously expressed, whereas *rho-1* is precisely spatially and temporally regulated [17,29], which has key influence on spatial transduction of the EGF signal. Therefore, to understand more precisely the mechanism through which RHO-1 regulates EGF signaling, an understanding of the regulatory mechanisms Rho-1 has on STAR is needed. Overall, Rho-1 is understood to be the primary effector of the EGF response, due to its ability to respond both to positive feedback and to induce EGF signaling in neighboring cells in oogenesis [25]. This mechanism is likely to be seen in embryonic development as well, exerting its effect through *spi* rather than *grk*. RHO-1 is a key point of cross-pathway regulation in early embryogenesis, which will be discussed below.

### 1.3. Transduction of the EGF Signal from the Receptor to ras

*Drosophila* was originally thought to have three homologs of vertebrate *ras*, a gene at 85D based on chromosome location (*ras85D* or *ras1*), the second gene at 64D (*ras64D* or *ras2*), and *rap1* (*ras3*) (which was later found to be a separate GTPase and not in fact a *ras* homolog). We will focus on Ras1 as it is most applicable to embryonic patterning [30,31]. *ras1* has been studied extensively in EGF—mediated signaling in the *Drosophila* eye and in higher organisms, and we will apply many of the structural findings which have elaborated the molecular mechanisms through which *ras1* transduces the EGF signal to the later components of the pathway in embryogenesis.

The basic outcome of EGF signal transduction is the activation of RAS. The actual mechanism is more complex. First, upon ligand binding, EGFR forms a dimer and *trans*-phosphorylates across the dimer. The protein Downstream of Receptor Kinase (DRK, the *Drosophila* homolog of mammalian Grb2) binds through its SH2 domain to the phosphorylated tyrosines on EGFR localizing it to the plasma membrane. In turn, DRK binds Son of Sevenless (SOS) the guanine exchange factor which triggers activation of RAS by promoting GTP binding [20,32].

RAS is therefore positively regulated by the EGF. It is also negatively regulated by *sprouty* (*sty*). *sty* was once thought to be an extracellular inhibitor of EGF signaling [33], but recent data suggests that *sty* functions as an intracellular inhibitor of the pathway modulating *ras* activity [34]. While the precise mechanism through which *sty* regulates the pathway is not clear, evidence suggests that STY interacts with DRK and GAP1, at least in eye development [14]. This interaction between STY and DRK was suggested to prevent the interactions of DRK with SOS and other accompanying activating factors. Understanding how *sty* functions will lead to a full molecular mechanism of signal transduction from EGF to *ras* [14].

### 1.4. Mechanism of RAS Activation of RAF and the Kinase Cascade

The main function of active RAS is to activate D-RAF (also known as *polehole* in *Drosophila* or just RAF), a highly conserved serine-threonine protein kinase. The fundamental function of activated RAS is to localize RAF to the plasma membrane, where RAF undergoes further activation [35,36]. RAF has three conserved regions, the first binds to RAS, the second is the negative regulatory domain, and the third is the kinase [37]. After RAF is activated by RAS, RAF phosphorylates mitogen-activated Protein kinase kinase (MAPKK, MKK), also known as also known as mitogen-activated protein kinase/extracellular signal-regulated kinase (ERK) Kinase (MEK). MEK was originally known in *Drosophila* as *downstream of raf-1* (*dsor1*), and was shown to have significant sequence similarity to murine MEK and Xenopus MAP Kinase activator. MEK was shown to be activated through direct phosphorylation by RAF, causing activation of kinase activity [38,39,40]. After MEK is activated by RAF it phosphorylates the *Drosophila* homolog of MAP kinase (MAPK)/extracellular signal regulated kinase (ERK), known as *rolled* [40,41]. Among many targets, activated *rolled* phosphorylates the transcription factors such as *pointed* and *yan* which in turn lead to transcriptional responses [42]. Cells with a loss of function in *rolled*, as expected, produce the same cell-death phenotype as seen in an EGF loss of function [43].

In embryogenesis, *pointed* is responsible for most of EGF’s effects on cell differentiation [44,45]. *pointed* has two promoters, allowing expression of two different transcript forms, which can in some cases show partial heteroallelic complementation [44]. A second *rolled* target is *yan* (also known as *anterior open*), a transcription factor responsible for inhibiting cell differentiation. In essence, the two transcription factors are in opposition with *pointed* as the activator of EGF pathway target genes and *yan* as the repressor.

**Figure 1:**
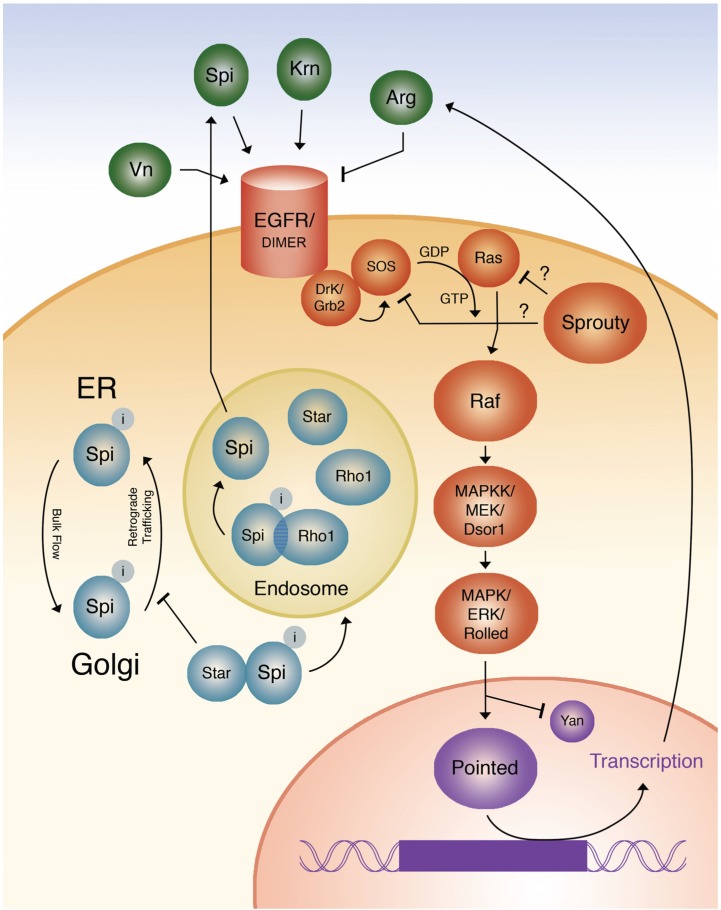
Diagrammatic representation of the EGF signaling pathway. Question marks refer to the still unknown mechanism of Sprouty action.

When the EGF signaling pathway is activated, *yan* is destabilized and therefore repressed, and *pointed* is activated [30]. The combined effect of *yan* derepression and *pointed* activation results in activation of EGF target genes such as *orthodenticle* (*otd*), *arg*, and *tartan* (which effect a wide variety of changes in different tissue [19]. Figure one summarizes the entire EGF signaling pathway (Figure 1).

## 2. Patterning of the Neuroectoderm

Three homeobox genes control the development of the dorsal-ventral axis of the *Drosophila* central nervous system: *muscle segment homeobox* (*msh*), *ventral nervous system defective* (*vnd*), and *intermediate neuroblasts defective* (*ind*) [46]. This subdivision is mediated by *toll*, *dpp*, and EGF signaling, which together are responsible for establishing three separate Hox domains. Specifically, where the *msh* domain is patterned due to repression of *dpp*, *ind* is expressed after being activated both by *Toll* and EGF signaling, and the *vnd* domain is established by *Toll* signaling and maintained by EGF signaling [46,47,48]. This process works through a repressive function of *dpp* signaling [49], and shows that EGF signaling works both to repress *dpp* signaling and to preserve the existing dorsal-ventral axis established by early *Toll* signaling [50,51,52]. While this model is highly compelling on a basic level, recent evidence has shown that development of the neuroectoderm depends on a vast web of connecting repressive and activating networks. Far from acting independently of one another, the three homeobox genes repress each other (through *ind* repression of *vnd*), and that EGF over expression causes the expansion of both *vnd* and *ind* into the lateral domain (originally *msh*-expressing domain) [53]. Recently, evidence has also emerged suggesting that EGF signaling is necessary for glial cell development, supporting the argument that EGF plays a more significant role in cell fate determination in the developing neuroectoderm. Kim et al. found that glial markers such as *gcm* and *repo* were reduced in the absence of EGF or *spi*, concluding that EGF signaling is responsible both for initial formation and maintenance of lateral glia. Likewise, over expressing EGF through over activated *spi* caused an expansion of the medial and intermediate cell fates into the lateral cell column, causing a repression of Repo [54]. This indicates that EGF has both necessary repressive and activating functions in specification of ventral nervous system cell fates, mediated by complex interactions with components of other signaling pathways. For example, while EGF was originally thought to only repress *dpp*, recent work shows that EGF could also be repressed by *dpp*. While *dpp* primarily functions at a short range, bounded by interaction with EGF and other pathways, *dpp* can also function indirectly at long-range in neuroblast development by repressing EGF signaling, again demonstrating the significant versatility of EGF signaling as a regulatory mechanism for precise spatial and temporal differentiation of the neuroectoderm [55].

Furthermore, we now know that EGF signaling has at least some degree of ability to pattern the neuroectoderm independently of its genetic effect on key neural development homeobox genes. EGF signaling has been shown to mediate the direct phosphorylation of *ind* through its downstream effector MAP kinase. Phosphorylation of *ind* results in repression of *ind* target genes, which allows EGF signaling to modify cell fate even in the presence of high levels of *ind* protein [56].

**Figure 2:**
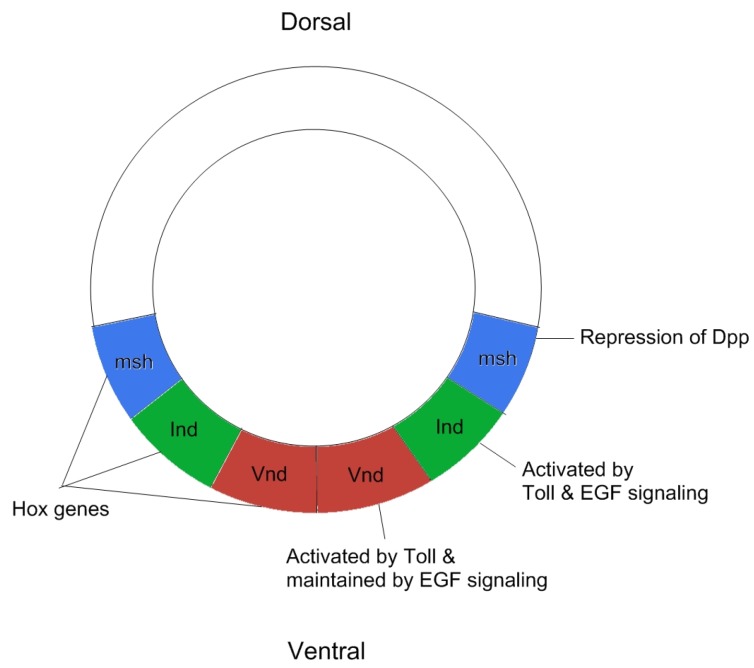
The signaling pathways involved in dorsal-ventral patterning of the neuroectoderm as shown by a transverse section of an early stage embryo (Stage 5 just before gastrulation starts).

This finding strengthens the conclusion that EGF signaling is necessary for neural development, as an active regulator of key homeobox genes and their protein products. This is further supported by the fact that the loss of EGF signaling results in widespread apoptosis of alternative neuroblast identities [57]. Finally, the regulatory mechanisms thought to be common to the diverse functions of the pathway (i.e., *argos*-mediated repression of EGF ligand binding) allows EGF signaling to maintain precise gradients of cell fate determination in the lateral regions of the ventral neuroectoderm [58]. Figure two summarizes the signaling events involved in patterning of the neuroectoderm (Figure 2).

## 3. Non-Neuroectodermal Ventral Patterning, Including Segmentation

EGF signaling is also involved in ventral epidermal patterning, where it interacts with *wnt* and *hedgehog* signaling in the patterning of denticles. There are two ways in which WNT and EGF signaling interact [59,60,61]. First, EGF signaling induces denticle cell fates through the transcription factor *shavenbaby* [62]. WNT signaling blocks EGF signaling and permits cells to develop the smooth cuticle cell fate [61,62], but in a complicated twist EGF is also required for the survival of smooth cuticle cells [61,63,64]. This is very similar to EGF signaling in the neuroectoderm where it is both a repressor of certain alternate neuroblast cell fates and requisite for the survival of most types of neuroblasts (see the above section). Second, *downstream of raf-1* (*Dsor1*, the *Drosophila* homolog of MEK1/2) has been shown to inhibit AXIN-mediated destruction of Armadillo (ARM) in an EGF signaling independent manner [60], demonstrating that there is crossover between these pathways independent of the outcome of EGF signaling. Third, EGF signaling has also been shown to link WNT signaling components with Toll signaling, by inducing expression of *wntD*, which limits *dorsal* (*dl*) nuclear localization at the poles and along the dorsal-ventral axis of the developing embryo [59]. Our recent findings further suggest that EGF signaling directly influences *toll* in embryonic patterning (unpublished observations). Overall, recent work on EGF signaling in non-neuroectodermal ventral patterning shows that EGF signaling is capable of playing a wide-ranging and diverse set of roles in its pathway interactions.

## 4. Specification of Muscle Precursors and Muscle Attachment to Tendons

Yarnitzky et al. showed in 1999 that *vein* (*vn*) and *spitz* (*spi*) are involved in the development of muscle precursors and argued that *vn* is qualitatively different from *spi* to avoid *arg*-mediated negative feedback into the EGF signaling process [64]. Unfortunately, since then few new insights have emerged into EGF’s function in developing muscle tissue in embryogenesis. The main recent findings: (1) the EGF pathway controls the specification and later maintenance of Adult Muscle Precursors [65]; and (2) that EGF signaling through *vn* and *spi* permits regenerative proliferation of multipoint gastric stem cells after damage [66]. Taken together, these findings both support the initial interpretation that *vn* differs qualitatively from *spi* in muscle precursor development [64]. More importantly, these findings provide significant evidence for an emerging theme in *Drosophila* EGF signaling: that most EGF mediated pathways in *Drosophila* embryogenesis are involved both in the initial establishment and subsequent maintenance of processes, as shown by the requirement for EGF in both repair and establishment of muscle tissue.

The multiple roles for EGF signaling necessitate the need for tight regulatory mechanisms ensuring the correct development of a complex organism, and at the same time, allowing flexibility in damage repair, and preventing aberrant growth. As many experiments in a variety of model organisms and in humans have shown, errors in EGF regulation result in highly tumorigenic growth, as the pathway’s excessive activation allows uncontrolled growth of many tissue types as well as metastasis [67].

## 5. Specification of Tracheal Invagination

A new focus for embryonic research is the effect of EGF signaling in tracheal development, specifically its role in specification of tissue integrity. EGF signaling was found to be necessary for maintaining tissue integrity of epithelial cells during tracheal development through modulation of cell adhesion a function independent of *pointed* one of the main transcription factors under the regulation of EGF [68]. Activation of EGF signaling leads to increased stiffness in epithelial tissue, and down-regulation of EGF signaling has the opposite effect [68], demonstrating a connection between EGF and Cadherin-modulated cell adhesion. The precise mechanism by which EGF modulates Cadherin based cell adhesion remains unclear, but a connection was suggested by another study which showed that in the absence of EGF signaling, developing tracheal cells are unable to concentrate filamentous Actin causing a defect in tracheal invagination [69]. EGF signaling was also shown to coordinate tracheal invagination by encircling the invagination site and promoting myosin cell intercalation [70]. The net result is a precise spatiotemporal activation sequence of EGF, which causes cells to invaginate as a group. Without EGF signaling, individual cells ingress without a clear coordinating strategy in the tissue, suggesting that EGF is crucial for specifying precise tissue boundaries in tracheal development [70,71]. Additionally, two receptor tyrosine phosphatases PTP10D and PTP4e negatively regulate EGF signaling during tracheal development [72]. Studies of tracheal development suggest that EGF provides very precise spatial and temporal information to cells in order to coordinate unified tissue development and movement.

## 6. Cell Recruitment to the Chordotonal Organs

Recent work has provided some additional insight into EGF’s influence on development of Chordotonal organs. Inbal et al. had previously shown that *atonal* (a transcription factor responsible for nerve cell development) triggers *rhomboid* expression, causing EGF activation, which initiates recruitment of attachment cells from the ectoderm and induce their specialization [73]. Again, it appears clear that EGF signaling is a common mechanism for precise and accurate tissue differentiation and maintenance of differentiation to prevent waste.

## 7. Specification of Oenocytes

In 2004, Brodu et al. [69] showed that oenocyte delamination occurs through discrete bursts, mediated by a localized EGF response. They found that EGF signaling triggered sequential activation of targets, which temporally communicated cell movement, a process which was continued by post-EGF mediated intracellular signaling [74]. Aside from this work, oenocyte specification has seen limited attention in the past 15 years.

## 8. Dorsal Midline Patterning (Including Dorsal Closure and Viability of Dorsal Midline Cells)

Antagonism between Dpp and EGF signaling is responsible for dorsal closure and corresponding patterning of cells near the dorsal midline [58]. Dpp blocks EGF signaling stimulating apoptosis near the midline [58]. This relationship is not unidirectional as EGF signaling down-regulates expression of Dpp in the epidermis, and prevents apoptosis in amnioserosa cells [75]. This signaling process highlights another common theme: EGF signaling is often required as the positive regulator of cell viability in key developmental processes, but also directly regulates the apoptosis gene Hid [76].

## 9. Other Signaling Pathways

EGF signaling is responsible for modulating square cell packing in the presumptive *Drosophila* pharynx by affecting planar cell polarity of cells in a square cell grid [77]. This process requires the effector Pins to orient mitotic spindles perpendicularly to the midline of the cell, which causes cells to adopt the square cell packed conformation [77]. This finding highlights the growing impact of EGF signaling on cell polarity.

## 10. Conclusions

### 10.1. Common Themes

(1) EGF often functions in cell growth and proliferation and regulates apoptosis.
(2) EGF signaling in *Drosophila* is highly self-regulating, particularly through RHO-1 and STAR mediated processing of EGF ligands and post-transcriptional control of ARGOS expression.
(3) EGF signaling also can differ qualitatively due to expression of different yet functionally similar ligands (i.e., *vein* vs. *spi*). The availability of ligands with slightly different functionality allows EGF signaling to be precisely regulated based on ligand availability, rather than attempting to modulate the effects of the pathway after activation by a single ligand.
(4) EGF signaling functions in cell adhesion and polarity. These four characteristics make EGF signaling highly effective at modulating tissue boundaries involving multiple alternative signaling pathways, as EGF signaling can self-regulate expression and interact with many other pathways through both negative and positive regulation.

### 10.2. Utility of Studying the EGF Signaling Pathway

Overall, EGF signaling is a crucial factor in promoting cell survival and differentiation. The study of EGF signaling in *Drosophila* embryogenesis has proven to be a valuable tool for understanding the complex mechanisms which give rise to the complexity of the various tissue systems of the *Drosophila* embryo. Careful study and analysis of EGF signaling mechanisms can reveal broader themes about embryonic control of cell fate, and are highly clinically applicable in understanding how aberrant EGF signaling can contribute to a wide range of human pathologies. This review did not cover other EGF pathways such as PI3K/PTEN, mTOR and Src as this has mainly been studied in larval and adult tissues. For EGF pathway studies, these tissues have provided a variety of useful tumor and drug screening models [78], aging models [79], colon stem cell models [80,81], brain cancer [82,83], and cell proliferation models [84].

With the advent of incredibly powerful new gene editing technologies such as the CRISPR/Cas9 system, and new mutants revealed in the genetic screens [85], there exists a plethora of new opportunities to study the pathway. New tools, such as live imaging in three dimensions and novel methods of fluorescent tagging, allow for the analysis of phenotypes which would otherwise be too ambiguous or complex to be studied through traditional means. In the WNT pathway, optogenetic approaches have been used to activate the co-receptor LRP6 (LDL receptor related protein, or Arrow in *Drosophila*) through a CRY2 fusion and blue light, an approach that should work for EGFR [76,86,87,88]. While much of the classic research on the pathway centered around *polehole* and the *faint little ball* phenotype, new methods will allow much more precise study of intracellular components of the pathway, and will allow a deeper understanding of the complex interactions between the EGF pathway and more well-studied embryonic signaling pathways, helping to further unravel the complexities of the developing *Drosophila* embryo. The work we have presented here represents a broad foundation upon which a deeper understanding of the mechanisms through which EGF signaling affects cancer can be built.
